# Can Infants Tell the Difference between Gold and Yellow?

**DOI:** 10.1371/journal.pone.0067535

**Published:** 2013-06-26

**Authors:** Jiale Yang, So Kanazawa, Masami K. Yamaguchi

**Affiliations:** Department of Psychology, Chuo University, Hachioji, Tokyo, Japan; University of Tokyo, Japan

## Abstract

There is a large literature focused on the color perception of matte surface. However, recent research showed that the component of surface specular reflection, such as glossiness, also affects categorical color perception. For instance, the color term “gold” was used to name high specular stimuli within a specific range of chromaticity, which overlaps with those of yellow and orange for low specular stimuli. In the present study, we investigated whether the component of surface specular reflectance affects the color perception of 5- to 8-month-old infants by using the preferential looking technique. In the first experiment, we conducted a simple test to determine whether infants perceive yellow and gold as the same color by comparing their preference for these colors over green. If the infants perceive yellow and gold as the same color, they would show similar preference scores over green. On the other hand, if infants show different preference scores over green, it indicates that infants do not perceive yellow and gold as the same color. Only the 7–8 month-old infants showed different preference scores for gold and yellow over green. This result indicates that the 7–8 month-old infants perceive gold and yellow as different colors. In Experiment 2, we eliminated the component of specular reflectance on the gold surface and presented it against green to infants. A similar preference score of yellow over green was obtained. This result suggests that the difference between the preference scores for gold and yellow over green in Experiment 1 was based on representations of glossiness.

## Introduction

Color perception is one of the fundamental aspects of human vision. Even in the immature vision system of early infancy, the red/green channel begins functioning around 2 months of age [1]–[6], and the blue/yellow channel does so by around 4 months of age [7]. Although the color spectrum is continuous, it isn’t necessary for humans to identify or memorize an infinite variation of colors because colors appear to be segmented into several discrete perceptual categories for adults [8], and infants [9]–[12].

Two kinds of surface reflectance are involved in the color perception of real objects: diffuse reflection and specular reflection. The diffuse reflection is distributed in every direction in a hemisphere over the surface, while the specular reflection is reflected into one outgoing direction only, which is defined by the law of reflection. Specular reflections produces sharp and bright patterns on the surface image and makes the surface look glossy, which contrasts with blurry shading patterns due to diffuse reflection. Previous studies of categorical color perception (e.g. [8]) had focused on this aspect of diffuse reflection; however, the latest research [13] shows that the component of specular reflection also affects categorical color perception. They used a categorical color naming task to test whether subjects’ color perception change following a change of the surface’s specular reflection. They found that color terms specific for metallic materials, namely, GOLD, SILVER, and COPPER, emerged as the specular reflectance of the stimuli increased, while use of the basic color terms, such as yellow, orange, and white, correspondingly declined. This result indicates that humans utilize the information from specular reflectance, such as surface gloss, to categorize surface colors.

The information from specular reflectance is also important for infants because it contains information on the attributes of the object’s surface. For instance, given the fact that most foods and drinks are wet and thereby glossy, the estimation of glossiness enables infants to recognize whether the stuff is eatable and drinkable. A recent study [14] found that 7- to 8-month-old infants perceive difference between glossy objects and matte objects based on the visual perception of a surface. This raises the possibility that the color perception of infants may be also affected by the specular reflectance of the surface. In the present study, we addressed the issue of whether infant color perception changes while the specular reflectance increases.

Investigating the color perception of infants poses an immense methodological challenge since, unlike adults, infants cannot report what color they perceive. Thus, it is difficult to test directly whether the color perception of infants changes. In the present study, we generated a set of object images by CG that have the same chromaticity but different specular reflectance, and compared whether infants’ preference for the images changed. Infants demonstrate different spontaneous preferences for different chromatic stimuli, for instance, they looked longest at ‘red’ and ‘blue’ and least at ‘green’ stimuli [15]–[19]. Therefore, if their preference of color changes, it provides suggestive evidence that the color perception of the infants has changed.

Previous research [17]–[19] about the color preferences of infants has shown that infants have a similar degree of preference for yellow and green. Furthermore, it happens that increasing the specular reflectance of a yellow surface can transform the surface’s appearance to gold; in contrast, this doesn’t occur with green. Therefore, these colors are appropriate for our investigation. In the first experiment, we conducted a simple test to determine whether infants perceive yellow and gold as the same color by comparing their preference for these colors over green. If the infants perceived yellow and gold as the same color, they would show identical preference scores for these colors over green. On the other hand, if they showed different preference scores over green, it would indicate that the infants did not perceive yellow and gold as the same color. In Experiment 2, we examine whether the infants’ discrimination between yellow and gold was based on representations of glossiness.

## Experiment 1

**Figure 1:**
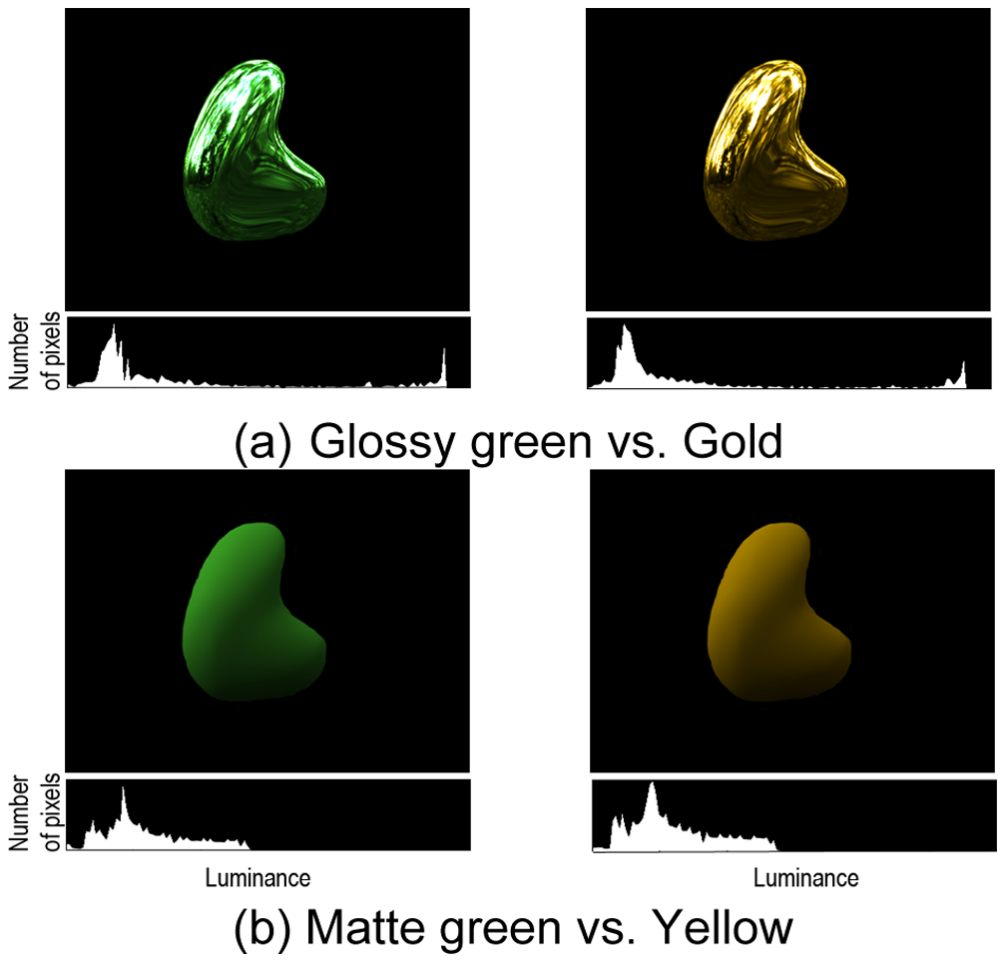
Stimuli used in Experiment 1. (a) glossy green vs. gold objects. (b) matte green vs. yellow objects. The pixel-luminance histogram (object region) is shown below each image.

In the first experiment, we investigated whether infants perceive yellow and gold as the same color by comparing their preference for these colors over green. Yang et al., (2012) [14] reported that surface glossiness affects infants’ preference for objects. Thus, it is necessary to control the surface glossiness of objects in an investigation of preference between yellow/gold over green. In terms of optics, glossiness is related to the specular reflection of the surface that faithfully mirrors the illumination environment. In this experiment, we used computer graphics software (NewTek LightWave 10.0) to generate images of objects which have identical specular reflections to control their surface glossiness. Four kinds of objects were generated (Fig. 1) : a gold object and a glossy green object, which have high specular reflection; a yellow object and a matte green object, which have low specular reflection. We presented these computer-generated images side by side on a CRT monitor, and observed which image the infants looked at longer during the fixed period of observation.

### Methods

#### Ethics statement

Ethical approval for this study was obtained from the ethical committee at the Japan Women’s University. Moreover, the experiments were conducted according to the principles laid down in the Helsinki declaration. Written informed consent was obtained from each infant’s parents prior to participation in the experiment.

#### Participants

Twelve infants aged 5–6 months (six male, six female, mean age = 160.4 days, ranging from 149 days to 188 days), and twelve infants aged 7–8 months (four male, eight female, mean age = 226.0 days, ranging from 201 days to 253 days) participated in the study. Although eight other infants were tested in our experiment, they were excluded from the analysis because of fussiness (n = 3), side bias of more than 90% (n = 2), or because they only looked at one side and did not compare the two images during one trial (n = 1). All infants were recruited by advertisements in the newspaper. All subjects were full-term at birth and healthy at the time of the experiment. Written informed consent was obtained from the parents of the participants.

#### Apparatus

The infant sat on his or her parent’s lap in the experimental booth during the experiment. In front of the infant, at a distance of about 40 cm, there was a 22-inch color CRT monitor that displayed all the stimuli. The resolution of the CRT was set at 1024×768 pixels with an 8-bit gray scale. The infant’s looking behavior was recorded through a video camera set under the monitor. Behind the experimental booth, the infant’s behavior was observed via a TV monitor.

#### Stimuli

We generated a set of visual stimuli that was defined in a three-dimensional space similar to the one previously used to examine the perception of gold [13], which was composed of a combination of the CIE xy chromaticity coordinates and diffuse/specular reflectance. The 3D shape was created (Figure 1) by the experimenters using LightWave Modeler 10.0 (NewTek). The glossy objects (the gloss green object and the gold object) had a specular reflectance of 0.9 and a diffuse reflectance of 0.1, while the matte objects (the matte green object and the yellow object) had a specular reflectance of 0.1 and a diffuse reflectance of 0.9. Because the averages of specular reflectance and diffuse reflectance for glossy objects and matte objects both are 0.5, thus the value of the specular reflectance would be used to indicate the combination of specular reflectance and diffuse reflectance. All images were rendered under a natural illumination field (Eucalyptus, Debevec 1998) using LightWave Layout 10.0 (NewTek). Because the natural scene used for the rendering contained varying chromaticities, the images had varying chromaticities. In order to compare the preference for clearly defined colors, we gave all the pixels in each stimulus the same chromaticity (yellow/gold: x = .47, y = .44; green: x = .36, y = .48, in CIE 1937), and the luminance values were unchanged. This image operation also had been used in the previous study [13] that investigated the categorical color properties of gold in adults. Each stimulus subtended 8×8 deg. The background was replaced by a uniform dark field of 0.51 cd/m^2^. The mean luminance within the object region was virtually equalized; 38.6 cd/m^2^ for the glossy object and 24.5 cd/m^2^ for the matte object, respectively. The glossy objects (the gloss green object and the gold object) had a higher contrast (SD/mean = 0.84) and skewness of luminance histogram (1.25) than the matte objects (the matte green object and the yellow object) (SD/mean = 0.53, skewness = 0.51).

#### Procedure

A preferential-looking paradigm was used in our experiment. Each infant was presented with two conditions (glossy green vs. gold objects and matte green vs. yellow objects) each of which contained two trials. In each trial, the stimuli were presented for 10 s. The position of the stimuli was reversed in two trials of the same condition. The order of the presentation was counterbalanced for each infant. In order to attract the infant’s attention, a fixation figure was shown in the center of the CRT monitor accompanied by a short beep sound prior to each trial. After confirming that the infant was looking at the fixation figure, the experimenter started the trial.

#### Data coding and analysis

An observer, who didn’t know the stimulus identity, measured the infant’s looking time based on an offline video movie. The observer recorded the infant’s looking time for the left or right presentation field by pressing one of two keys when the infant was looking at the relevant field. When the infant looked away from the presentation field, no recording was made. The video camera’s sample rate was 60 Hz.

### Results and Discussion

**Figure 2:**
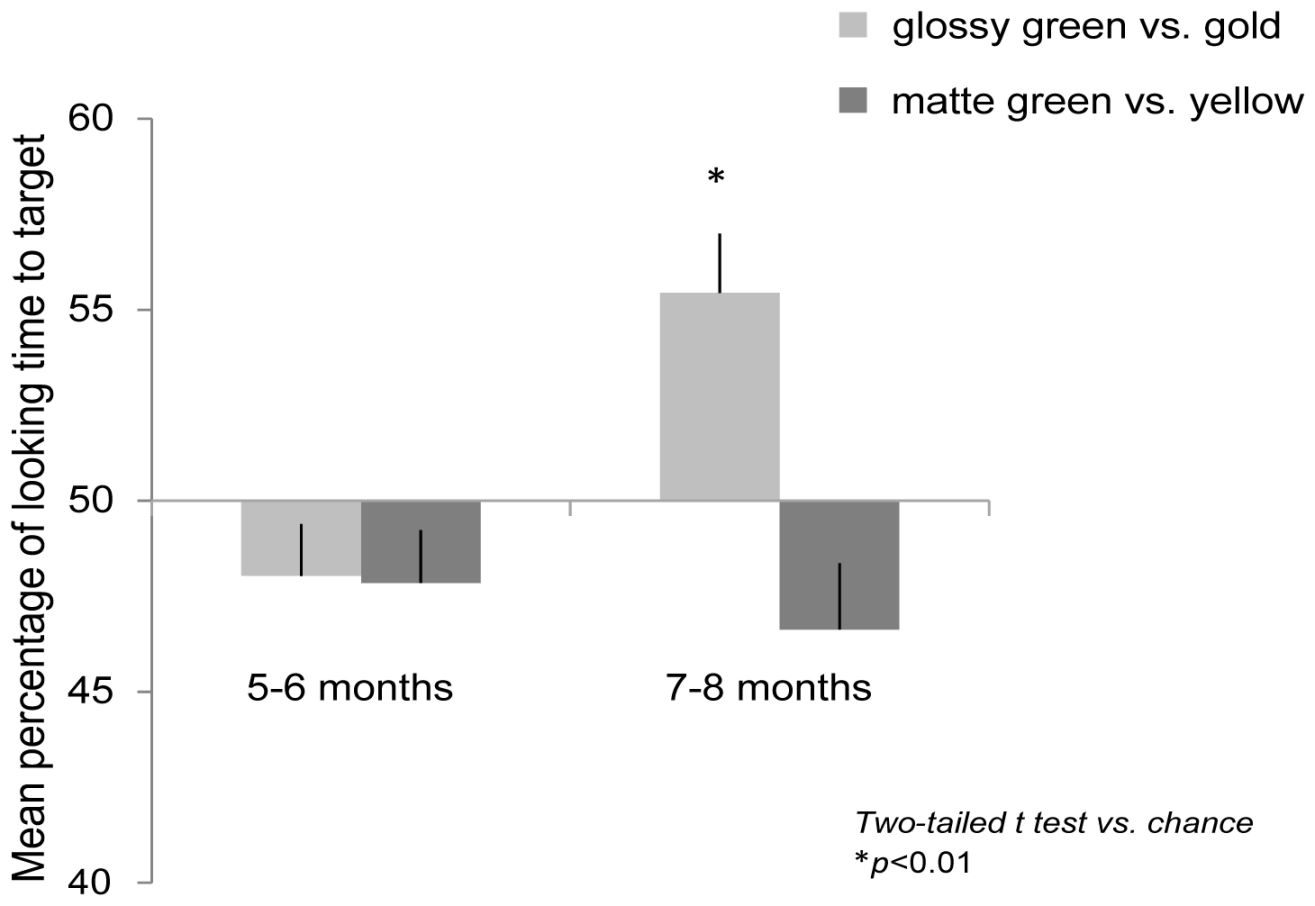
Result of Experiment 1. Mean percentage of looking time to target. Error bars are +1 standard error of the mean. The result of glossy green vs. gold is indicated by the light gray bar, and the result of matte green vs. yellow is indicated by the dark gray bar.

In the condition of glossy green vs. gold, the mean total looking time of the two trials was 13.5 s for 5- to 6-month-olds (67.6% of total trial duration), and 12.2 s for 7- to 8-month-olds (60.8% of total trial duration). We calculated the percentage of looking time for the gold object (which we will refer to as target) for each infant. In Fig. 2, the light gray bars show the mean percentage of glossy green vs. gold. A two-tailed t-test with chance (50%) revealed that 7- to 8-month-olds showed preference for the gold object over the glossy green object (*t*(11) = 3.55, *p<.01*), but not 5- to 6-month-olds (*t*(11) = 1.69, *n.s.*).

In the condition of the matte green vs. yellow objects, the mean total looking time of the two trials was 9.6 s for 5- to 6-month-olds (47.9% of total trial duration), and 11.0 s for 7- to 8-month-olds (55.1% of total trial duration). We calculated the percentage of looking time for the yellow object (which we will refer to as target) for each infant. In Fig. 2, the dark gray bars show the mean percentage of matte green vs. yellow. The two-tailed t-test showed that both 5- to 6-month-olds (*t*(11) = 1.97, *n.s.*) and 7- to 8-month-olds (*t*(11) = 1.95, *n.s.*) didn’t show preference for the yellow object.

Only the 7–8 month old infants showed a change in looking preferences for glossy yellow vs. glossy green, when compared to matte yellow vs. matte green. This result indicates that the 7- to 8-month-old infants perceive gold and yellow as different colors, even though the gold and yellow had identical chromaticities. Adult research [13] has shown that the color terms GOLD and SILVER are categorical color terms specifically associated with glossy surfaces. Therefore, perceiving surface glossiness is necessary for infants to discriminate between gold and yellow. If infants couldn’t perceive the surface glossiness of the objects, their preference for the gold object shown in Experiment 1 would disappear. In the next experiment, we tested this possibility.

## Experiment 2

**Figure 3:**
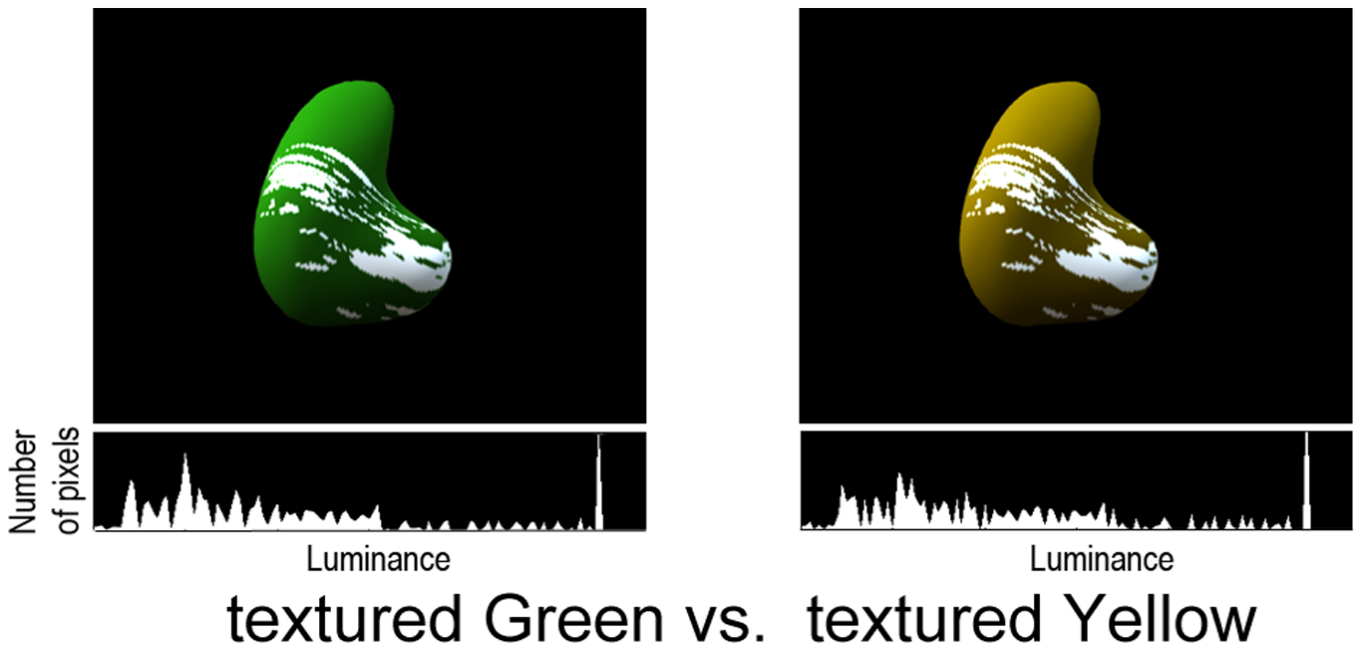
Stimuli used in Experiment 2. The textured objects have similar contrast and luminance histogram to the glossy object image in Experiment 1, but look matte. The pixel-luminance histogram (object region) is shown below each image.

In the Experiment 2, we examine whether the infants’ discrimination between yellow and gold was based on representations of glossiness. We introduced a pair of new stimuli shown in Fig. 3, in which a matte surface is covered with albedo texture-like white paint splashes. These objects have a similar contrast and skewness of luminance histogram to the glossy object image, but look matte. If the difference of preference scores for gold and yellow against green in Experiment 1 was based on representations of glossiness, they would show similar preference scores to that of the condition of matte green vs. yellow of Experiment 1. On the other hand, if the difference of preference scores showed in Experiment 1 was based on low-level image statistics, they would show similar preference scores to that of the condition of glossy green vs. gold of Experiment 1.

### Methods

#### Participants

Twelve infants aged 7–8 months (four male, eight female, mean age = 224.6 days, ranging from 205 days to 247 days) participated in the study. Although four other infants were tested in our experiment, they were excluded from the analysis because of fussiness (n = 2), side bias of more than 90% (n = 1), or because they looked only at one side of the figure during one trial (n = 1).

#### Stimuli

The textured object was made by mapping an albedo texture on to the matte surface. The albedo texture was the binarized (and rotated) image of the specular-reflection pattern (highlights) in the glossy object. The texture was mapped as increments of diffuse reflection along the 3D structure of the matte surface. The relative intensity of the texture (splashes) was adjusted so that the contrast and skewness of luminance histogram were as close to the glossy object as possible. The resulting image of the textured object had a contrast (SD/mean) of 0.89, skewness of 1.12 and mean luminance of 36.5 cd/m^2^, which were similar to those of the glossy object.

#### Apparatus and procedure

The apparatus were the same as those used in Experiments 1. Each infant was presented with two trials (textured yellow vs. textured green object) in which the position of the stimuli was reversed. In each trial, the stimuli were presented for 10 s. The order of the presentation was counterbalanced for each infant.

### Results and Discussion

**Figure 4:**
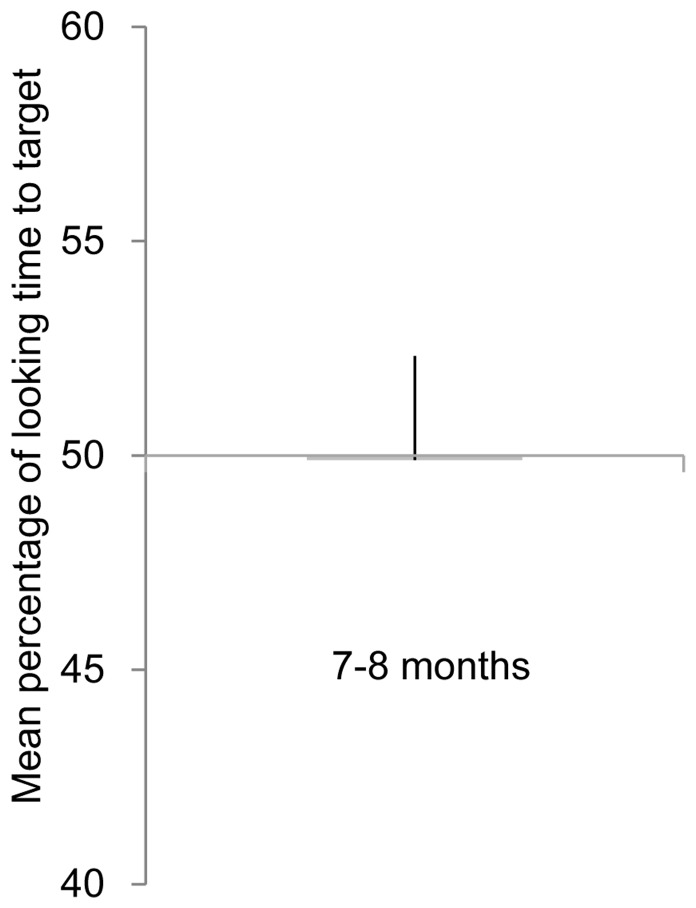
Result of Experiment 1. Mean percentage of looking time to target. Error bars are +1 standard error of the mean.

A two-tailed t-test with chance (50%) was performed to examine whether the infants showed any preference. As in the condition of matte green vs. yellow of Experiment 1, the infants showed no preference for the textured yellow object (*t*(11) = 0.04, *n.s.*) to the textured green object (Fig. 4). We used an independent samples t-test to examine whether this preference was different from that in the condition of matte green vs. yellow in Experiment 1, and no significant difference has been shown (*t*(22) = 2.07, *n.s.*). This result suggests that difference of preference scores for gold and yellow against green in Experiment 1 was based on representations of glossiness.

## General Discussion

The present study investigated whether the component of surface specular reflectance affects color perception among 5- to 8-month-old infants by using the preferential looking technique. In Experiment 1, we investigated whether infants perceived the yellow object and gold object as the same color by comparing their preference for each over a green object. If infants showed different preference scores over green, it would indicate that the infants do not perceive yellow and gold as the same color. The results showed that only the 7- to 8-month-old infants showed different preference scores for gold and yellow against green. This result indicates that the 7- to 8-month-old infants perceive gold and yellow as different colors, even though gold and yellow had identical chromaticities. In Experiment 2, we confirmed that this discrimination between gold and yellow was not based on an image statistic, such as contrast and skewness, but probably based on the surface glossiness. These findings suggest that 7- to 8- month-old infants distinguish surfaces based on their specular reflectance, as do adults.

Our finding that 7- to 8-month-old infants use surface gloss to distinguish surface colors is in accordance with our previous study [14] which showed the emergence of the perception of glossiness occurs around 7 to 8 months of age. Furthermore, the age found in this present study is consistent with the emergence of discriminating various attributes of objects and surfaces such as shape from shading (7 months: [20]–[22]), motion from shadows (7 months: [23]–[24]), and transparency (4 to 7 months: [25]–[27]).In terms of the development of color perception, it is suggested that infants can discriminate the color and lightness of an image in the spatial context at around 4- to 5-months -old [28]–[31], but cannot use them to identify an object until 6 to 7 months of age [32]–[33]. These synchronous developments imply a common level behind developmental neural processes underlying the perception of object properties, including glossiness. It is known that in adults the perception of each of these properties often depends upon the perception of the others [34]–[37]. Our findings indicate a possibility that such cross-modular neuronal networks develop around 7 to 8 months of age.

The first wave of developmental research in object perception focused on shape and color (e.g. [32]–[33]). However, natural objects possess a variety of attributes beyond just shape and color. An object’s materials are just as important as its shape and color [39]. Our study is the first finding to show interaction between the perception of material surface properties, such as glossiness, and the perception of color even in infancy. This indicates that infants may be able to distinguish different materials. Future studies, for instance, a direct test of cross-modal interactions between touch and vision using real objects, are needed to explore the origin of the perception of surface material. In the present study, we have shown that surface specular reflectance affects the color perception of infants; but there are numerous other surface attributes, such as transparency [40]–[41] or texture [42], that can affect color perception. A deeper understanding of the developmental relationship between the perception of surface material and color awaits models that account for how these perceptions interact with one another as well as with object shape, object pose, and illumination geometry.

In present study, we found a change in looking preference for glossy yellow vs. glossy green, when compared to matte yellow vs. matte green. This result indicated that infants’ color perception would be affected not only by chromaticities, but also by surface specular reflectance. Although the change of looking preferences could be explained by perceiving the difference between metallic green and matte green, a more plausible explanation is that the infants could discriminate the categorical difference between ‘Gold’ and ‘Yellow’, because gold-colored materials are more common than green metals. This hypothesis is consistence with the evidences for categorical responding to color in pre-linguistic infants [9]–[12] and chimpanzee [38], which showed that even infant and primates have some biological foundation of categorical color perception innately. Further research may be able to find the evidence to support this hypothesis by using another method, such as habituation paradigm.
